# Evaluation of Serum Proinflammatory Cytokine IL-17A and Tight Junction Protein Claudin-1 in Psoriasis

**DOI:** 10.1155/2022/6092808

**Published:** 2022-03-07

**Authors:** Lina Xu, Yunlei Pan, Shunli Tang, Juan Bai, Yinhua Wu, Jianjun Qiao, Hong Fang

**Affiliations:** Department of Dermatology, The First Affiliated Hospital, College of Medicine, Zhejiang University, Hangzhou 310003, China

## Abstract

**Objective:**

This study aimed to estimate serum IL-17A and Claudin-1 levels, investigate their correlation, and evaluate their diagnostic significance as potential blood-based biomarkers in psoriasis.

**Methods:**

Serum IL-17A and Claudin-1 concentrations were determined using enzyme-linked immunosorbent assay (ELISA). Statistical analyses were performed to determine differences in serum levels of IL-17A and Claudin-1, their bivariable correlation with psoriasis severity, as Psoriasis Area Severity Index (PASI), and their predictive abilities using receiver operating characteristic (ROC) curves.

**Results:**

Significantly higher IL-17A and lower Claudin-1 levels were found in psoriasis (*p* < 0.05). PASI did not correlate significantly with either IL-17A or Claudin-1 in psoriasis and their subtypes. The only significant correlation between serum IL-17A and Claudin-1 was shown in late-onset psoriasis (*r* *=* *0*.*630*, *p* *=* *0*.*028*). ROC curve analysis indicated the serum IL-17A, serum Claudin-1, and combination of IL-17A and serum Claudin-1 for predicting psoriasis with the areas under the curve (AUC) of 0.951 (*p* < 0.0001), 0.709 (*p* = 0.0119), and 0.949 (*p* < 0.0001), respectively. Moreover, the potential role in distinguishing between early-onset and late-onset psoriasis: we obtained serum IL-17A, serum Claudin-1, and their combination AUC of 0.590 (*p* = 0.3126), 0.741 (*p* = 0.0045), and 0.741 (*p* = 0.0067), respectively. However, none of the serum IL-17A, serum Claudin-1, and their combination was well-performed discriminating mild psoriasis from moderate-to-severe psoriasis with AUC of 0.553 (*p* = 0.5596), 0.518 (*p* = 0.8539), and 0.559 (*p* = 0.5225), respectively.

**Conclusion:**

These preliminary results suggest that the serum Claudin-1 as a potential biomarker and the relationship and possible regulatory interactions between IL-17A and Claudin-1 in psoriasis are distinguishable by age of onset.

## 1. Introduction

Psoriasis is a multifactorial chronic, immune-mediated, inflammatory, systemic dermatosis of complex pathogenesis including genetic, environmental, and immunological factors [1]. The classic psoriatic morphology of erythematous scaly plaques is histopathologically due to hyperkeratosis, parakeratosis, acanthosis, angiogenesis, and perivascular lymphohistiocytic infiltrate. Moreover, psoriasis coexists with both physical and psychiatric multimorbidity, which leads to high disease burden, morbidity, and mortality [2]. It approximately affects 60 million adults worldwide and cannot currently be cured [3]. Immunological mechanism of the centrality of IL-17A, the shooting target of the therapeutic “hierarchy,” have been identified as key drivers of psoriasis pathogenesis and psoriasis multimorbidity [4]. Thus, biologics targeting IL-17A and its receptor have currently achieved revolutionary success therefore [5]. However, the inconsistency of targeting IL-17A antibodies poses a formidable problem.

As is well known, psoriasis has long been associated with skin barrier dysregulation [6], in which tight junction (TJ), as an important component in epidermal barrier function, is also seen to be clearly altered in psoriasis [7,8]. Claudin-1, a composition of TJ proteins that are extremely important to skin barrier, is reported to be downregulated in the uppermost and lowermost layers in early stages of skin lesion [9–11], while in the late stages of skin lesion, it is often completely absent [10]. Until now, there is little consensus on Claudin-1 patterns in psoriatic skin. Besides, the significance of serum Claudin-1 in psoriasis is not known yet. Therefore, the etiopathogenesis of psoriasis still needs to be studied.

The aim of the current investigation was to estimate serum IL-17A and Claudin-1 levels in psoriasis, detect their correlation with disease severity, and examine whether they are blood-based candidate biomarkers associated with psoriasis and subtypes.

## 2. Materials and Methods

### 2.1. Patients

This study comprised 43 consecutively selected psoriatic patients, along with 16 age- and gender-matched healthy subjects as controls, at our dermatology clinics between May 2017 and December 2018. All psoriatic patients were clinically diagnosed by two experienced dermatologists. Patients without a history of psoriasis but having any other chronic-inflammatory, allergic, autoimmunological, genetic, metabolic, or neoplastic diseases, which might interfere with the outcome of psoriasis, were excluded. All controls were enrolled from physical examinees without a family history of psoriasis and not having a history of taking any kind of medication within the past three months. Information on age, gender, age at onset, skin psoriasis duration, family history, body mass index (BMI), and body surface area (BSA) were recorded. Psoriasis Area Severity Index (PASI) was performed by a certified dermatologist. The severity of psoriasis was classified as mild psoriasis (BSA <3% or PASI <5) and moderate-to-severe psoriasis (BSA ≥3% or PASI ≥5). The psoriasis was also classified as early-onset psoriasis (age at onset <40 years) and late-onset psoriasis (age at onset >40 years); all patients signed their written informed consent.

### 2.2. Methods

Venous blood samples (5 ml) were collected by venipuncture without anticoagulant under sterile conditions from all patients and controls. Within 30 minutes, all samples were rapidly centrifuged for 5 minutes at 1000 rpm and then immediately stored at −80°C until the assays were processed. Serum levels of IL-17A and Claudin-1 were determined by solid-phase sandwich ELISA (LifeSpan BioSciences, Inc., Seattle, WA). All assays were conducted based on manufacturers' protocols. These assays were only applicable for humans and as per manufacturers' instructions, the minimum detectable dose was 1 pg/mL for IL-17A (catalogue number: LS-F27021) and 0.1 ng/mL for Claudin-1 (catalogue number: LS-F20989) at 450 nm.

### 2.3. Statistical Analysis

The statistical analysis was performed using the IBM SPSS Statistics for Windows, version 22 (IBM Corp., Armonk, N.Y., USA). Presentation graphics were made using GraphPad Prism 5.0 (GraphPad software, San Diego, CA). A *p* value <0.05 was considered statistically significant. The study population's characteristics were analyzed by descriptive statistics. The normality of continuous variables was tested using the Shapiro–Wilk test and were presented as means ± standard deviations (SD), or medians (ranging from minimum to maximum values). Comparisons among groups were made using one-way ANOVA Kruskal–Wallis tests. Correlations were determined using Spearman's coefficient of rank correlation between Claudin-1, IL-17A, PASI, and other clinical characteristics (age, gender, age at onset, disease duration, family history, comorbidities, BSA, BMI, IL-2, IL-4, IL-6, IL-10, TNF-*α*, IFR-*γ*, CRP, and IgE). ROC curve analysis of the IL-17A and Claudin-1 assays was conducted to evaluate the diagnostic detection performance of psoriasis.

## 3. Results

A total of 43 psoriatic patients, 29 males (67.44%) and 14 (32.59%) females, and 16 healthy control subjects were enrolled into this study (Supplementary Table 1). There were no statistical differences between patients and controls for age and gender (*p* > 0.05). The mean age in psoriatic patients was 40.42 ± 16.19 years (range 13–77 years), their mean age at onset was 31.74 ± 16.25 years (range 10–73 years), and the mean duration of psoriasis was 8.82 ± 8.80 years (range 0.25–30 years). Of the 43 psoriatic patients, 8 (18.6%) had a family history of psoriasis. The mean PASI was 3.62 ± 3.06 (range 0.20–15.20).

### 3.1. Differential Expression of Serum IL-17A and Claudin-1 in Psoriasis

**Figure 1:**
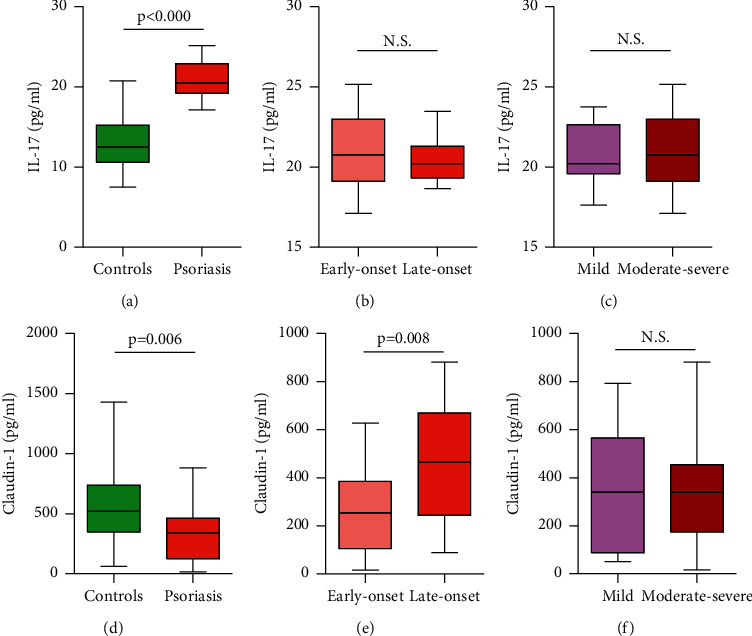
IL-17A and Claudin-1 levels in psoriasis. ELISA was utilized to detect serum IL-17A levels in (a) psoriasis and controls, (b) early- and late-onset psoriasis, and (c) mild and moderate-to-severe psoriasis. Serum Claudin-1 levels were compared between (d) psoriasis and controls, (e) early- and late-onset psoriasis, and (f) mild and moderate-to-severe psoriasis.

The results of comparative analysis on serum levels of IL-17A and Claudin-1 are presented in Supplementary Table 2 and Figure 1, and show a significant difference between the psoriatic patients (IL-17A: 20.88 ± 0.31, range 17.12–25.16; Claudin-1: 327.00 ± 33.00, range 15.98–880.01, respectively) and healthy controls (IL-17A: 13.12 ± 0.88, range 7.52–20.76; Claudin-1: 538.82 ± 85.12, range 60.62–1428.69, respectively) with the Mann–Whitney *U* test (IL-17A, *p* ≤ 0.001; Claudin-1, *p* = 0.006; Figures 1(a) and 1(d)). Serum Claudin-1 (but not IL-17A) levels show a highly significant difference (*p* = 0.008) between early-onset and late-onset psoriasis patients (Figures 1(b) and 1(e) and Supplementary Table 3). There was no significant difference between serum IL-17A and Claudin-1 levels (*p* = 0.576 and *p* = 0.760, respectively) in patients with mild and moderate-to-severe psoriasis (Figures 1(c) and 1(f) and Supplementary Table 3).

### 3.2. Relationship between Serum IL-17A, Claudin-1, and PASI Scores in Psoriasis

**Figure 2:**
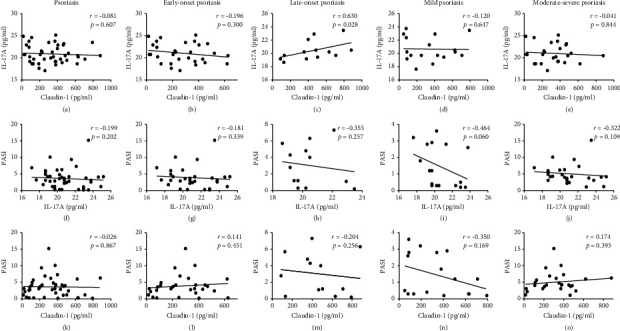
Correlation between IL-17A, Claudin-1, and PASI score. Spearman's correlation analysis was applied to test the correlation between IL-17A and Claudin-1 in (a) psoriasis, (b) early-onset psoriasis, (c) late-onset psoriasis, (d) mild psoriasis, and (e) moderate-to-severe psoriasis, respectively; the correlation between PASI and IL-17A in (f) psoriasis, (g) early-onset psoriasis, (h) late-onset psoriasis, (i) mild psoriasis, and (j) moderate-to-severe psoriasis; and the correlation between PASI and Claudin-1 in (k) psoriasis, (l) early-onset psoriasis, (m) late-onset psoriasis, (n) mild psoriasis, and (o) moderate-to-severe psoriasis.

Bivariate correlation analysis suggested serum IL-17A significantly correlated with Claudin-1 only in subtypes of late-onset psoriasis (*r* = 0.630, *p* = 0.028), but not of early-onset psoriasis (*r* = − 0.196, *p* = 0.300), mild psoriasis (*r* = − 0.120, *p* = 0.647), and moderate-to-severe psoriasis (*r* = − 0.041, *p* = 0.844), as shown in Table S4. However, PASI was significantly correlated with neither IL-17A nor Claudin-1 in psoriasis (*r* = − 0.199, *p* = 0.202; *r* = − 0.026, *p* = 0.867, respectively), in early-onset psoriasis (*r* = − 0.181, *p* = 0.339; *r* = 0.141, *p* = 0.451, respectively), in late-onset psoriasis (*r* = − 0.355, *p* = 0.257; *r* = − 0.026, *p* = 0.526, respectively), in mild psoriasis (*r* = − 0.464, *p* = 0.060; *r* = − 0.350, *p* = 0.169, respectively) and in moderate-to-severe psoriasis (*r* = − 0.322, *p* = 0.109; *r* = 0.174, *p* = 0.395, respectively) in Figure 2 and Supplementary Table 4.

**Figure 3:**
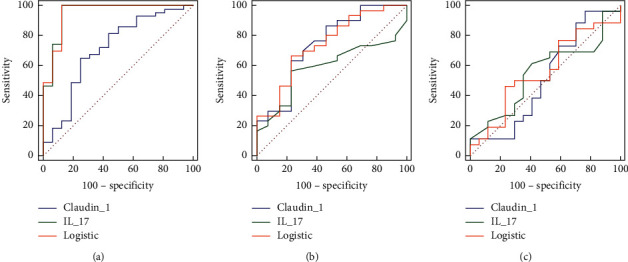
Discriminators of ROC curves for serum IL-17A and Claudin-1 alone or combined and potential biomarkers of psoriasis and its subtypes. ROC curves for the obtained key discriminators of serum IL-17A, serum Claudin-1 alone, and their combination (logistic). (a) Psoriasis vs. controls; (b) early- vs. late-onset psoriasis; (c) mild- vs. moderate-severe psoriasis.

*Diagnostic Ability of the Serum IL-17A and Claudin-1 in Psoriasis.* ROC curves were subsequently constructed to evaluate the performance of IL-17A and Claudin-1 in psoriasis (as shown in Supplementary Table 5 and Figure 3). The combination of IL-17A and Claudin-1 exhibited equal performance in discriminating psoriasis from controls (AUC: 0.949, *p* < 0.0001), with the model comprising only IL-17A (AUC: 0.951, *p* < 0.0001), which performed better than the model including just Claudin-1 (AUC: 0.709, *p* = 0.0119). Remarkably, the combination of IL-17A and Claudin-1 (AUC: 0.741, *p* = 0.0067) almost performed identically to Claudin-1 alone (AUC: 0.741, *p* = 0.0045), while IL-17A (AUC: 0.590, *p* = 0.3126) had poor performance separating early-onset psoriasis from late-onset psoriasis (Supplementary Table 5 and Figure 3(b)). However, none of the models, IL-17A alone (AUC: 0.553, *p* = 0.5596) or just Claudin-1 (AUC: 0.518, *p* = 0.8539) or their combination (AUC: 0.559, *p* = 0.5225), was shown to have better discriminatory performance to discriminate mild psoriasis from moderate-to-severe psoriasis (Supplementary Table 5 and Figure 3(c)).

## 4. Discussion

In the literature, several studies have appeared investigating serum levels of IL-17A in psoriasis and controls and the results reported are controversial. In line with our results, some studies presented a significant increase in the serum levels of IL-17A in psoriatic patients compared to controls [12–20]. However, other studies reached the opposite conclusion [21–30]. Moreover, contradictory results have also been observed for correlation between IL-17A and PASI. Our study showed no correlation of IL-17A with PASI in psoriasis, which was consistent with some [17, 23, 24, 28] and inconsistent with other previous studies [12, 13, 15, 18, 19]. Neither in early- or late-onset psoriasis nor in mild or moderate-to-severe psoriasis, we did not yet find more of their correlation. Moreover, ROC curves analysis showed outstanding performance of serum Claudin-1 for separating early-psoriasis from late-onset psoriasis. The combinatorial biomarkers of serum IL-17A and Claudin-1 may serve as a good predictor for psoriasis, while they each or combined could not be candidate biomarkers assessing psoriasis severity.

Clearly, there is some disputation regarding the results on serum IL-17A levels and its correlation with disease severity assessed by PASI in psoriasis. The reasons probably might be the heterogeneity of the study populations and inclusion criteria (Table 1). The elevated levels of serum IL-17A may potentially be mediated by psoriatic skin, joint, nail, and comorbidities, while PASI is a relatively simple indicator of severity just for skin [21]. When the condition of psoriasis becomes complex, it is difficult to explain serum levels of IL-17A by the emerging complications. Therefore, given this consideration, the correlation found between the disease severity indicator on skin (PASI) and any serum cytokine (including IL-17A) levels might also be deceptive. Consequently, subgrouping studies of cytokines (including IL-17A) and PASI might provide more valid results in psoriatic patients only with tortured skin rather than that beyond skin lesions. This might partly explain the reason for our selecting outpatients, psoriatic patients.

To our knowledge, this is the first study investigating serum Claudin-1 in psoriasis. Currently, studies regarding Claudin-1 in psoriasis have been focused on ex vivo experiments of human or animal skin and in vitro experiments of keratinocytes. However, its researches on circulating levels are lacking, which might provide more and be a new angle to psoriasis, an essentially chronic-systemic-inflammatory dermatosis. The possible reasons could be that dermatologists focus too much on its skin-specific expression in dermatoses and thus ignore and miss the evaluation of serum level of Claudin-1. In our current study, combinatorial biomarkers of serum IL-17A and Claudin-1 are potential predictive markers for psoriasis and Claudin-1 for identification onsets of psoriasis, although little is known about the role of Claudin-1 in the age at onset. The two subtypes, early- and late-onset psoriasis, are respectively associated with different multiple comorbidities, psychosocial impact, and immunological profiles due to different genetic backgrounds in psoriasis with different ages of onset [31]. Presumably, different genetic backgrounds might significantly affect or be affected by Claudin-1. Further research is warranted on the potential relationship between ages of onset and Claudin-1. On the other hand, correlation between PASI and serum levels of Claudin-1 should be performed more reliably in psoriatic patients only with tortured skin than that beyond skin lesions, similar to relationship between PASI and IL-17A.

We found that the association of serum IL-17A and Claudin-1 was significant in early/late-onset psoriasis; however, inconsistently, some studies reported no difference and others a significant difference in several other diseases, in animal models of other diseases and in cellular models of other diseases. The question whether IL-17A regulates Claudin-1 remains unanswered; findings that they are correlated should be met with caution: further prospective studies are required, especially in psoriasis. Therefore, we speculate on the role of IL-17A on Claudin-1, which may contribute to new therapeutic approaches.

The limitations of our study are as follows: first, most of our patients enrolled with lower PASI scores. Despite the statistically significant difference, it was quite clear that few higher PASI score patients were enrolled, potentially biasing our results. Therefore, we are tremendously interested in learning the results of analyses with patients with higher PASI scores. Second, PASI itself may have some limitations, having been criticized for being laborious, complex, insensitive, low-specific, and nonlinear for reflecting the degree of improvement or worsening in psoriasis [32]. However, in reality, the PASI score remains the most extensively used clinical severity score in psoriasis, the most thoroughly validated method, and the most commonly used clinical means to evaluate the severity of psoriasis [33]. Consequently, our conclusions are inevitably somewhat controversial using the lower PASI score patients enrolled and the nonlinear PASI severity scale.

## 5. Conclusions

Despite the biologic agents targeting IL-17A and its receptor having well-evidenced therapeutic efficacy, our results found that higher levels of serum IL-17A do not correlate with psoriasis severity as represented by PASI score, neither in early-versus late-onset psoriasis nor in mild versus moderate-to-severe psoriasis. Notably, both serum IL-17A and Claudin-1 do not correlate with PASI in psoriasis and in its subgroups, except for correlation in late-onset psoriasis. Previous studies have investigated Claudin-1 in skin, but serum Claudin-1 levels remained unknown in psoriasis, while our results remind us of serum Claudin-1 as a potentially sensitive diagnostic marker for distinguishing early-onset psoriasis from late-onset psoriasis. Rather promisingly, new data on the roles of IL-17A and Claudin-1 and on the regulation of Claudin-1 by IL-17A in psoriasis may indicate new therapeutic approaches.

## Figures and Tables

**Table 1:** Inclusion criteria and populations' characteristics of the published studies conducted to evaluate the levels of IL-17A in the serum of psoriatic patients.

Study populations' characteristics/inclusion criteria									
Ref. number	Age (years)	Sex (F/M)	Psoriasis forms	Nail psoriasis	Psoriatic arthritis	Comorbidities	PASI (0–72)	IL-17A (pg/ml)	Treatment
[13]	Mean ± SD: 47.5 ± 7.6 Range: 25.0–72.0	41/81	Psoriasis vulgaris: 102/122	N/A	Present in 8/122	N/A	Mean ± SD:7.3 ± 4.2 Range: 0.7–32.3	Mean ± SD: 8.3 ± 3.8 Range:0.0–13.9	Treated, untreated, well-controlled, and poorly controlled cases. Treatments: topical steroid, topical vitamin D3, psoralen ultraviolet A, and systemic treatments (etretinate, ciclosporin)
			Guttate psoriasis: 7/122						
			Erythrodermic psoriasis: 5/122						
[12]	N/A	N/A	Moderate-severe plaque psoriasis	N/A	Excluded	N/A	N/A	N/A	Untreated topically or systematically ≥1 month before enrolment
[14]	N/A	20/14	Plaque psoriasis	N/A	N/A	N/A	N/A	Median: 6.9 Range: 5.3–8.8	N/A
[16]	Mean ± SD: 43.9 ± 15.2 Range: 18.0–71.0	21/27	Plaque psoriasis	N/A	N/A	N/A	N/A	Mean ± SD: 10.5 ± 60.4	Untreated topically (apart from emollients) and systematically ≥4 weeks before enrolment
[21]	Mean ± SD: 35.0 ± 15.5 Range: 7.0–79.0	12/18	Plaque psoriasis	N/A	N/A	N/A	Mean ± SD: 9.3 ± 8.2 Range: 1.5–33.3	Mean ± SD: 8.3 ± 3.8 Range: 0.0–13.9	Untreated topically or systematically ≥2 months before enrolment
[15]	Mean ± SD: 39.9 ± 14.9	34/36	Plaque psoriasis: 30	N/A	N/A	N/A	Mean ± SD: 6.6 ± 5.4	Mean ± SD: 6.6 ± 8.0	Newly diagnosed or without systemic treatment ≥2 months before enrolment
			Guttate psoriasis: 20						
			Pustular psoriasis: 20						
[22]	Mean ± SD: 45.6 ± 13.2 Range: 18.0–69.0	10/50	Psoriatic patients	N/A	N/A	N/A	Mean ± SD: 15.7 ± 9.7 Range: 4.8–64.2	Mean ± SD: 3.2 ± 1.8	N/A
[23]	Mean ± SD: 44.5 ± 15.6 Range: 23.0–72.0	23/9	Active, chronic plaque psoriasis	Excluded	Excluded	N/A	Mean ± SD: 5.2 ± 3.7 Range: 2.2–15.0	Mean ± SD: 2.7 ± 3.5 Range: 0.0–8.6	Untreated topically and systematically ≥1 year before enrolment
[24]	Mean ± SD: 43.7 ± 12.4 Range: 20.0–60.0	30/22	Plaque psoriasis	Present in 12/52	Excluded	N/A	Mean ± SD:4.6 ± 1.9 Range: 2.1–9.0	Mean ± SD: 4.3 ± 6.2 Range: 0.0–28.5	Untreated topically or systematically ≥1 year before enrolment
[25]	Mean ± SD: 44.8 ± 15.2		Plaque psoriasis	N/A	N/A	N/A	N/A	Range: 38.8–9.0	N/A
[17]	Mean ± SD: 50.2 ± 13.3	24/30	Plaque psoriasis	N/A	N/A	N/A	Median: 16.4 Range: 7.0–41	Median: 3.9 Range: 3.9–3530	N/A
[26]	Mean ± SD: 36.6 ± 14.2	N/A	Plaque psoriasis: 38	N/A	N/A	N/A	Mean ± SD: 8.2 ± 4.0	Mean ± SD: 1.4 ± 2.4	Untreated topically or systematically ≥4 weeks before enrolment
			Guttate psoriasis: 30						
[27]	Mean: 51.8 Range: 50.0–70.0	20/0	Pustular palmoplantar psoriasis	N/A	N/A	N/A	N/A	Mean ± SD: 2.0 ± 4.13 Range: 0.0–17.1	Untreated topically ≥2 weeks and systematically ≥4 months before enrolment
[28]	Mean ± SD: 29.7 ± 13.5	7/27	Plaque psoriasis	N/A	N/A	N/A	Mean ± SD: 4.9 ± 3.0 Range: 0.5–11.4	N/A	Untreated topically ≥2 weeks and systematically ≥8 weeks before enrolment
[18]	Mean: 37.0 Range: 16.0–65.0	13/17	Plaque psoriasis	N/A	N/A	N/A	Mean ± SD: 20.5 ± 8.5	Mean ± SD: 2.5 ± 1.7	Untreated topically ≥6 weeks and systematically ≥3 months before enrolment
[19]	Median: 38.8	0/8	Plaque psoriasis	N/A	N/A	N/A	N/A	Median: 0.5	N/A
[20]	N/A	2/3	N/A	N/A	N/A	N/A	N/A	Mean ± SD: 0.7 ± 0.1	N/A
[29]	>18	24/36	Severe psoriasis	N/A	Present in 26/70	Present in 30/60	N/A	Mean ± SD: 2.6 ± 3.2 Range: 1.8–3.4	N/A
[30]	Mean: 42.1 ± 15.0	12/28	Severe plaque psoriasis	N/A	N/A	N/A	Mean ± SD: 9.8 ± 6.3	N/A	N/A
Our study	Mean ± SD: 31.8 ± 16.2 Range: 10–73	14/29	Plaque psoriasis	Present in 32/43	Present in 8/43	Present in 32/43	Mean ± SD: 3.6 ± 3.0 Range: 0.2–15.2	Mean ± SD: 20.9 ± 0.3 Range: 17.1–25.2	Treated, untreated, well-controlled, and poorly controlled cases. Treatments: topical steroid, topical vitamin D3, psoralen ultraviolet A, and systemic treatments (etretinate, ciclosporin)

Ref. no.: reference number; F/M: females/males; N/A: not available; ^∗^data retrieved from the abstract, since no full-text file was available. N/A, not available.

## Data Availability

The data used to support the findings of this study are available from the corresponding author upon request.

## Supplementary Materials

Supplementary Materials Supplementary Table 1: patients' demographic and clinical characteristics, as well as IL-17A and Claudin-1 serum levels. Supplementary Table 2: the values of serum assay IL-17A and Claudin-1 in psoriatic patients and controls. Supplementary Table 3: patients' demographic and clinical characteristics, as well as the serum levels of IL-17A and Claudin-1. Supplementary Table 4: correlation between serum IL-17A, Claudin-1, and other parameters in the psoriasis group and subgroups. Supplementary Table 5: diagnostic performances of serum IL-17A, Claudin-1, and IL-17A + Claudin-1 in psoriasis. *Supplementary Material*.
