# Supplementary material for: Evaluation of Serum Proinflammatory Cytokine IL-17A and Tight Junction Protein Claudin-1 in Psoriasis
Source: Contrast Media Mol Imaging. 2022 Mar 7;2022:6092808. doi: 10.1155/2022/6092808 (PMC8920681; doi:10.1155/2022/6092808)
Supplement: Supplementary Materials — Supplementary Table 1: patients' demographic and clinical characteristics, as well as IL-17A and Claudin-1 serum levels. Supplementary Table 2: the values of serum assay IL-17A and Claudin-1 in psoriatic patients and controls. Supplementary Table 3: patients' demographic and clinical characteristics, as well as the serum levels of IL-17A and Claudin-1. Supplementary Table 4: correlation between serum IL-17A, Claudin-1, and other parameters in the psoriasis group and subgroups. Supplementary Table 5: diagnostic performances of serum IL-17A, Claudin-1, and IL-17A + Claudin-1 in psoriasis. Supplementary Material. [file 6092808.f1.zip › 6092808.f1/supplementary TABLE (1).docx]

| Supplementary Table 1: Patients’ demographic and clinical characteristics, as well as IL-17A and claudin-1 serum levels. | | | | | | | | | | |
| --- | --- | --- | --- | --- | --- | --- | --- | --- | --- | --- |
| Psoriatic patients (n=43) | | | | | | | | | Controls(n=16) | |
| Patient number | Age (years) | Gender | Age at onset (years) | Disease duration (years) | Family history | PASI (0–72) | Claudin-1 (pg/mL) | IL-17 A(pg/mL) | Claudin-1 (pg/mL) | IL-17 A(pg/mL) |
| 1 | 44 | Male | 20.00 | 20.00 | No | 6.90 | 258.32 | 17.12 | 546.84 | 7.52 |
| 2 | 26 | Male | 23.00 | 3.00 | No | 4.90 | 113.14 | 18.66 | 401.30 | 10.04 |
| 3 | 43 | Male | 26.00 | 17.00 | No | 3.10 | 188.80 | 18.66 | 679.96 | 9.60 |
| 4 | 61 | Male | 44.00 | 17.00 | Yes | 0.30 | 131.83 | 19.70 | 791.86 | 12.28 |
| 5 | 31 | Male | 21.00 | 10.00 | No | 10.10 | 373.31 | 19.18 | 1428.69 | 13.68 |
| 6 | 59 | Male | 59.00 | 0.25 | No | 1.20 | 707.58 | 19.70 | 331.09 | 10.47 |
| 7 | 41 | Female | 11.00 | 30.00 | No | 3.60 | 86.71 | 20.23 | 437.04 | 11.82 |
| 8 | 31 | Male | 20.00 | 11.00 | No | 0.40 | 249.88 | 19.70 | 856.05 | 10.92 |
| 9 | 77 | Female | 73.00 | 4.00 | No | 4.30 | 379.18 | 19.18 | 755.24 | 12.74 |
| 10 | 48 | Female | 40.00 | 8.00 | Yes | 5.70 | 126.48 | 18.66 | 125.14 | 11.37 |
| 11 | 42 | Female | 37.00 | 5.00 | No | 3.20 | 191.55 | 17.63 | 386.53 | 13.21 |
| 12 | 55 | Female | 50.00 | 5.00 | No | 4.80 | 352.50 | 20.23 | 559.28 | 14.16 |
| 13 | 20 | Female | 10.00 | 10.00 | Yes | 2.90 | 432.55 | 19.70 | 491.45 | 15.62 |
| 14 | 34 | Male | 27.00 | 7.00 | No | 3.10 | 22.25 | 20.76 | 676.73 | 20.76 |
| 15 | 54 | Female | 34.00 | 20.00 | Yes | 3.70 | 205.32 | 20.76 | 93.29 | 16.62 |
| 16 | 25 | Male | 20.00 | 5.00 | No | 6.20 | 323.87 | 19.97 | 60.62 | 19.18 |
| 17 | 28 | Male | 12.00 | 16.00 | No | 0.30 | 627.01 | 20.50 | - | - |
| 18 | 45 | Male | 30.00 | 15.00 | No | 15.20 | 300.89 | 23.48 | - | - |
| 19 | 26 | Male | 24.00 | 2.00 | No | 4.20 | 226.11 | 21.30 | - | - |
| 20 | 66 | Male | 51.00 | 15.00 | Yes | 7.30 | 398.34 | 22.11 | - | - |
| 21 | 25 | Male | 24.00 | 1.00 | No | 2.80 | 339.77 | 21.30 | - | - |
| 22 | 45 | Male | 25.00 | 20.00 | Yes | 9.40 | 219.16 | 21.57 | - | - |
| 23 | 44 | Male | 22.00 | 22.00 | No | 2.40 | 522.10 | 21.03 | - | - |
| 24 | 46 | Male | 43.00 | 3.00 | No | 6.30 | 880.01 | 20.50 | - | - |
| 25 | 15 | Female | 13.00 | 2.00 | No | 4.10 | 109.15 | 24.60 | - | - |
| 26 | 26 | Male | 14.00 | 12.00 | Yes | 0.20 | 404.26 | 22.93 | - | - |
| 27 | 45 | Female | 41.00 | 4.00 | No | 0.20 | 791.86 | 23.48 | - | - |
| 28 | 31 | Male | 23.00 | 8.00 | No | 3.80 | 470.18 | 23.21 | - | - |
| 29 | 73 | Female | 69.00 | 4.00 | No | 1.10 | 464.13 | 22.93 | - | - |
| 30 | 37 | Male | 36.50 | 0.50 | No | 1.20 | 15.98 | 24.88 | - | - |
| 31 | 57 | Male | 30.00 | 27.00 | Yes | 2.20 | 32.38 | 20.76 | - | - |
| 32 | 27 | Male | 24.00 | 3.00 | No | 6.00 | 620.65 | 18.66 | - | - |
| 33 | 37 | Male | 36.50 | 0.50 | No | 0.30 | 631.78 | 20.23 | - | - |
| 34 | 17 | Male | 15.00 | 2.00 | No | 0.30 | 80.16 | 22.38 | - | - |
| 35 | 45 | Male | 35.00 | 20.00 | No | 0.50 | 50.29 | 22.93 | - | - |
| 36 | 13 | Male | 12.50 | 0.50 | No | 2.60 | 78.85 | 23.76 | - | - |
| 37 | 42 | Female | 27.00 | 15.00 | No | 3.10 | 370.38 | 23.76 | - | - |
| 38 | 24 | Male | 23.00 | 1.00 | No | 4.20 | 379.18 | 25.16 | - | - |
| 39 | 44 | Female | 43.00 | 1.00 | No | 2.80 | 88.02 | 19.18 | - | - |
| 40 | 18 | Female | 16.00 | 2.00 | No | 1.80 | 379.18 | 18.40 | - | - |
| 41 | 63 | Male | 55.00 | 8.00 | No | 4.00 | 485.36 | 20.50 | - | - |
| 42 | 38 | Male | 37.50 | 0.50 | No | 4.20 | 450.55 | 18.92 | - | - |
| 43 | 70 | Female | 68.00 | 2.00 | Yes | 1.20 | 503.68 | 19.44 | - | - |

| Supplementary Table 2.The values of serum assay IL-17A and claudin-1 in psoriatic patients and controls. | | | |
| --- | --- | --- | --- |
| Assay | Psoriatic patients (n=43) | Controls （n=16) | p values |
| Claudin-1(pg/mL) |  |  |  |
| Mean±SD | 327.00±33.00 | 538.82±85.12 |  |
| Median (min–max) | 339.78 (15.98-880.01) | 519.15 (60.62-1428.69) | **0.0063**** |
| IL-17A (pg/ml) |  |  |  |
| Mean±SD | 20.88±0.31 | 13.12±0.88 |  |
| Median (min–max) | 20.50 (17.12-25.16) | 12.51 (7.52-20.76) | ＜0.0001*** |
| Mann-Whitney U test; **P < 0.001, ***P <0.0001. | | | |

| Supplementary Table 3.Patients’ demographic and clinical characteristics, as well as the serum levels of IL-17A and clauidn-1. | | | | | | | |
| --- | --- | --- | --- | --- | --- | --- | --- |
| Characteristics | Psoriasis | Early-onset psoriasis ((n=30) | Late-onset psoriasis (n=13) | *p* values | Mild psoriasis (n=17) | Moderate-severe psoriasis (n=26) | *p* values |
| Age (year) | 40.42±16.19 | 33.13±11.54 | 57.23±12.52 | **≤0.001***** | 36.59±16.53 | 42.92±15.78 | 0.214 |
| Gender (Female/Male) | 29/14 | 23/7 | 6/7 | 0.051 | 10/7 | 19/7 | 0.341 |
| Early/late onset (<40/>40) | 30/13 | 30 | 13 | - | 11/6 | 19/7 | 0.57 |
| Mild/moderate-severe psoriasis | 17/26 | 1.63±0.49 | 1.54±0.42 | 0.57 | 9/8 | 16/10 | - |
| Stages (Active/stable) | 25/18 | 16/14 | 9/4 | 0.344 | 17 | 26 | 0.587 |
| Disease duration (years) | 8.82±8.10 | 10.25±8.75 | 1.31±0.48 | 0.078 | 7.90±8.68 | 9.42±7.82 | 0.552 |
| Family history (Yes/No) | 9/34 | 5/25 | 4/9 | 0.308 | 9/43 | 34/43 | 0.742 |
| BSA (%) | 11.24±19.59 | 11.03±16.17 | 11.74±26.65 | 0.915 | 0.95±0.64 | 17.97±22.92 | **0.004**** |
| BMI ( kg/m^2^) | 24.25±3.32 | 23.63±3.43 | 25.68±2.66 | 0.062 | 23.40±2.90 | 24.80±3.52 | 0.18 |
| claudin-1 (pg/mL) | 327.04±216.42 | 270.73±175.54 | 456.98±251.49 | **0.008**** | 339.70±246.05 | 318.76±199.41 | 0.760 |
| IL-17A (pg/mL) | 20.88±2.04 | 21.06±2.23 | 20.45±1.49 | 0.37 | 20.66±1.83 | 21.02±2.19 | 0.576 |
| IL-2 (pg/mL) | 0.75±1.51 | 0.36±0.94 | 1.06±2.35 | 0.166 | 0.24±0.47 | 0.79±1.89 | 0.243 |
| IL-4 (pg/mL) | 0.15±0.24 | 0.11±0.75 | 0.22±0.43 | 0.182 | 0.10±0.00 | 0.18±0.31 | 0.315 |
| IL-6 (pg/mL) | 26.29±74.34 | 16.46±31.02 | 48.98±127.34 | 0.191 | 16.25±22.35 | 32.86±94.08 | 0.48 |
| IL-10 (pg/mL) | 0.85±2.87 | 0.45±0.93 | 1.77±5.04 | 0.171 | 1.51±4.51 | 4.82±0.56 | 0.227 |
| TNF-α (pg/mL) | 1.70±2.78 | 1.26±2.06 | 2.73±3.90 | 0.113 | 1.34±2.22 | 1.94±3.16 | 0.494 |
| IFN-γ (pg/mL) | 0.21±0.37 | 0.18±0.34 | 0.27±0.44 | 0.454 | 0.21±0.45 | 0.21±0.32 | 0.999 |
| CRP (pg/mL) | 4.20±7.18 | 4.85±8.39 | 2.70±2.57 | 0.371 | 1.93±2.31 | 5.69±8.79 | 0.093 |
| IgE (pg/mL) | 115.62±183.66 | 128.00±146.93 | 84.66±115.26 | 0.367 | 110.78±154.72 | 118.91±129.84 | 0.855 |
| Nail psoriasis (Yes/No) | 23/20 | 15/15 | 8/5 | 0.498 | 11/6 | 17/9 | 0.055 |
| Arthritis (Yes/No) | 8/35 | 5/25 | 3/10 | 0.63 | 14/3 | 4/22 | 0.514 |
| Comorbidity (Yes/No) | 32/11 | 10/20 | 12/1 | 0.08 | 15/2 | 17/9 | 0.097 |
| PASI (0–72) | 3.63±3.03 | 3.89±3.23 | 3.04±2.50 | 0.406 | 1.45±1.26 | 5.06±3.00 | **≤0.001***** |
| **P < 0.001, ***P <0.0001. | | | | | | | |

| Supplementary Table 4. Correlation between serum IL-17A, claudin-1 and other parameters in the psoriasis group and subgroups. | | | | | | | | | | |
| --- | --- | --- | --- | --- | --- | --- | --- | --- | --- | --- |
| Characteristics | Psoriasis (n=43) | | Early-onset psoriasis (n=30) | | Late-onset psoriasis (n=13) | | Mild psoriasis (n=17) | | Moderate-severe psoriasis(n=26) | |
|  | IL-17A | Claudin-1 | IL-17A | Claudin-1 | IL-17A | Claudin-1 | IL-17A | Claudin-1 | IL-17A | Claudin-1 |
|  | r(*p*) | r(*p*) | r(*p*) | r(*p*) | r(*p*) | r(*p*) | r(*p*) | r(*p*) | r(*p*) | r(*p*) |
| Age (year) | -0.131(0.401) | 0.178(0.254) | -0.118(0.536) | -0.166(0.382) | -0.081(0.802) | -0.126(0.697) | -0.290(0.259) | 0.231(0.373) | 0.193(0.345) | 0.246(0.226) |
| Gender (Female/Male) | -0.144(0.356) | -0.004(0.980) | -0.114(0.549) | -0.027(0.886) | -0.247(0.440) | -0.416(0.178) | -0.516(**0.034***) | 0.122(0.641) | -0.104(0.613) | -0.075(0.715) |
| Early/late onset (<40/>40) | -0.143(0.360) | 0.384(**0.011***) | - | - | - | - | -0.215(0.407) | 0.452(0.068) | 0.370(0.063) | 0.410(**0.037***) |
| Mild/moderate-severe | 0.090(0.565) | -0.031(0.845) | 0.084(0.659) | 0.016(0.933) | 0.097(0.764) | -0.145(0.653) | - | - | - | - |
| Stages (Active/stable) | -0.049(0.753) | -0.034(0.828) | -0.189(0.316) | 0.054(0.777) | 0.258(0.418) | -0.051(0.874) | 0.291(0.257) | -0.048(0.855) | -0.063(0.759) | 0.242(0.233) |
| Disease duration (years) | -0.027(0.865) | -0.16(0.306) | -0.175(0.355) | -0.010(0.960) | 0.048(0.882) | -0.448(0.144) | 0.082(0.753) | -0.269(0.296) | -0.080(0.696) | -0.078(0.707) |
| Family history (Yes/No) | -0.030(0.849) | -0.069(0.660) | 0.047(0.807) | -0.005(0.978) | 0.130(0.687) | 0.028(0.931) | -0.128(0.624) | 0.170(0.515) | -0.293(0.147) | 0.072(0.728) |
| BSA (%) | -0.004(0.979) | 0.073(0.644) | -0.065(0.732) | 0.160(0.398) | -0.505(0.094) | -0.362(0.247) | 0.387(0.125) | 0.136(0.603) | 0.148(0.470) | 0.049(0.811) |
| BMI ( kg/m^2^) | 0.011(0.946) | 0.173(0.267) | 0.024(0.899) | 0.126(0.506) | 0.123(0.703) | -0.056(0.863) | 0.331(0.195) | -0.034(0.896) | 0.087(0.671) | 0.021(0.921) |
| Claudin-1 (pg/mL) | -0.081(0.607) | 1.000(≤0.001) | -0.196(0.300) | 1.000(≤0.001) | 0.630(**0.028***) | 1.000(≤0.001) | -0.120(0.647) | 1.000(≤0.001) | -0.041(0.844) | 1.000(≤0.001) |
| IL-17A (pg/mL) | 1.000(≤0.001) | -0.081(0.607) | 1.000(≤0.001) | -0.196(0.300) | 1.000(≤0.001) | 0.630(**0.028***) | 1.000(≤0.001) | -0.120(0.647) | 1.000(≤0.001) | -0.041(0.844) |
| IL-2 (pg/mL) | -0.036(0.819) | 0.177(0.256) | 0.104(0.586) | 0.278(0.137) | -0.098(0.762) | 0.047(0.885) | 0.148(0.572) | 0.162(0.535) | -0.041(0.844) | -0.106(0.607) |
| IL-4 (pg/mL) | -0.178(0.252) | -0.009(0.957) | -0.161(0.395) | 0.118(0.535) | -0.609(0.036*) | -0.371(0.235) | NS | NS | 0.267(0.187) | 0.062(0.762) |
| IL-6 (pg/mL) | -0.397(**0.008****) | 0.024(0.881) | -0.324(0.080) | 0.034(0.859) | -0.674(0.016*) | -0.113(0.727) | -0.513(**0.035***) | 0.267(0.301) | -0.002(0.993) | 0.178(0.384) |
| IL-10 (pg/mL) | -0.015(0.925) | -0.032(0.841) | 0.177(0.349) | -0.128(0.499) | -0.376(0.228) | 0.036(0.911) | 0.055(0.834) | -0.075(0.776) | -0.094(0.649) | 0.018(0.929) |
| TNF-α (pg/mL) | -0.425(**0.005****) | 0.204(0.189) | -0.364(**0.048***) | 0.206(0.275) | -0.342(0.276) | 0.299(0.346) | -0.383(0.129) | 0.306(0.232) | 0.009(0.965) | -0.041(0.841) |
| IFN-γ (pg/mL) | 0.129(0.410) | -0.128(0.412) | 0.183(0.333) | -0.201(0.287) | -0.352(0.262) | -0.131(0.685) | 0.026(0.922) | -0.255(0.323) | 0.128(0.533) | 0.289(0.153) |
| CRP (pg/mL) | -0.063(0.686) | 0.056(0.720) | -0.081(0.671) | 0.149(0.432) | 0.007(0.983) | -0.392(0.208) | -0.141(0.588) | 0.097(0.711) | -0.014(0.946) | 0.045(0.826) |
| IgE (pg/mL) | 0.149(0.340) | 0.089(0.571) | 0.148(0.435) | 0.153(0.419) | 0.039(0.905) | 0.252(0.430) | -0.065(0.803) | -0.047(0.859) | 0.014(0.944) | 0.003(0.988) |
| Nail psoriasis (Yes/No) | -0.246(0.111) | 0.068(0.666) | -0.185(0.327) | 0.154(0.416) | -0.493(0.103) | -0.318(0.313) | -0.367(0.147) | 0.025(0.924) | 0.178(0.385) | 0.210(0.303) |
| Arthritis (Yes/No) | 0.034(0.830) | 0.12(0.442) | 0.191(0.311) | 0.036(0.849) | -0.421(0.173) | 0.195(0.543) | -0.200(0.442) | 0.396(0.115) | -0.178(0.385) | -0.441(**0.024***) |
| Comorbidity (Yes/No) | -0.144(0.357) | -0.026(0.870) | -0.147(0.437) | -0.127(0.505) | -0.264(0.407) | -0.480(0.114) | -0.225(0.385) | 0.112(0.669) | -0.065(0.754) | 0.275(0.174) |
| PASI (0–72) | -0.199(0.202) | -0.026(0.867) | -0.181(0.339) | 0.141(0.451) | -0.355(0.257) | -0.204(0.526) | -0.464(0.060) | -0.350(0.169) | -0.322(0.109) | 0.174(0.395) |
| r, Spearman’s correlation coefficient; *p*, significance; *, p<0.05; **, p<0.01; NS, not available. | | | | | | | | | | |

| Supplementary Table 5.Diagnostic performances of serum IL-17A, claudin-1 and IL-17A+ claudin-1 in psoriasis. | | | | | | | | |
| --- | --- | --- | --- | --- | --- | --- | --- | --- |
| Group | Method | Cut-off | Sensitivity(95%CI) | Specificity(95%CI) | LR+ | LR- | AUC(95%CI) | P value |
| Psoriasis/controls | IL-17A | 16.620 | 100.00%(91.8%-100.0%) | 87.50%(61.7%-98.4%) | 8.00 | 0.00 | 0.951(0.861-0.990) | **<0.0001***** |
|  | Claudin-1 | 379.178 | 65.12%(49.1%-79.0%) | 75.00%(47.6%-92.7%) | 2.60 | 0.47 | 0.709(0.577-0.820) | **0.0119*** |
|  | Combination | 0.498 | 100.00%(91.8%-100.0%) | 87.50%(61.7%-98.4%) | 8.00 | 0.00 | 0.949(0.858-0.989) | **<0.0001***** |
| Early/late-onset psoriasis | IL-17A | 20.496 | 76.92%(46.2%-95.0%) | 56.67%(37.4%-74.5%) | 1.78 | 0.41 | 0.590(0.429±0.737) | 0.3126 |
|  | Claudin-1 | 450.553 | 53.85%(25.1%-80.8%) | 86.67%(69.3%-96.2%) | 4.04 | 0.53 | 0.741(0.585±0.862) | **0.0067**** |
|  | Combination | 0.263 | 76.92%(46.2%-95.0%) | 66.67%(47.2%-82.7%) | 2.31 | 0.35 | 0.741(0.585±0.862 | **0.0045**** |
| Mild/moderate-severe psoriasis | IL-17A | 20.230 | 61.54%(40.6%-79.8%) | 58.82%(32.9%-81.6%) | 1.49 | 0.65 | 0.553(0.394-0.705) | 0.5596 |
|  | Claudin-1 | 620.645 | 96.15%(80.4%-99.9%) | 23.53%(6.8%-49.9%) | 1.26 | 0.16 | 0.518(0.361-0.673) | 0.8539 |
|  | Combination | 0.615 | 46.15%(26.6%-66.6%) | 70.59%(44.0%-89.7%) | 1.57 | 0.76 | 0.559(0.399-0.710) | 0.5225 |
| *, p<0.05;**, p<0.01; ***<0.001 | | | | | | | | |
