# Supplementary material for: Evaluation of Serum Proinflammatory Cytokine IL-17A and Tight Junction Protein Claudin-1 in Psoriasis
Source: Contrast Media Mol Imaging. 2022 Mar 7;2022:6092808. doi: 10.1155/2022/6092808 (PMC8920681; doi:10.1155/2022/6092808)
Supplement: Supplementary Materials — Supplementary Table 1: patients' demographic and clinical characteristics, as well as IL-17A and Claudin-1 serum levels. Supplementary Table 2: the values of serum assay IL-17A and Claudin-1 in psoriatic patients and controls. Supplementary Table 3: patients' demographic and clinical characteristics, as well as the serum levels of IL-17A and Claudin-1. Supplementary Table 4: correlation between serum IL-17A, Claudin-1, and other parameters in the psoriasis group and subgroups. Supplementary Table 5: diagnostic performances of serum IL-17A, Claudin-1, and IL-17A + Claudin-1 in psoriasis. Supplementary Material. [file 6092808.f1.zip › 6092808.f1/Supplementary Table 1 (1).doc]

| Supplementary Table 1: Patients’ demographic and clinical characteristics, as well as IL-17A and claudin-1 serum levels. | | | | | | | | | | |
| --- | --- | --- | --- | --- | --- | --- | --- | --- | --- | --- |
| Psoriatic patients (n=43) | | | | | | | | | Controls(n=16) | |
| Patient number | Age (years) | Gender | Age at onset (years) | Disease duration (years) | Family history | PASI (0–72) | Claudin-1 (pg/mL) | IL-17 A(pg/mL) | Claudin-1 (pg/mL) | IL-17 A(pg/mL) |
| 1 | 44 | Male | 20.00 | 20.00 | No | 6.90 | 258.32 | 17.12 | 546.84 | 7.52 |
| 2 | 26 | Male | 23.00 | 3.00 | No | 4.90 | 113.14 | 18.66 | 401.30 | 10.04 |
| 3 | 43 | Male | 26.00 | 17.00 | No | 3.10 | 188.80 | 18.66 | 679.96 | 9.60 |
| 4 | 61 | Male | 44.00 | 17.00 | Yes | 0.30 | 131.83 | 19.70 | 791.86 | 12.28 |
| 5 | 31 | Male | 21.00 | 10.00 | No | 10.10 | 373.31 | 19.18 | 1428.69 | 13.68 |
| 6 | 59 | Male | 59.00 | 0.25 | No | 1.20 | 707.58 | 19.70 | 331.09 | 10.47 |
| 7 | 41 | Female | 11.00 | 30.00 | No | 3.60 | 86.71 | 20.23 | 437.04 | 11.82 |
| 8 | 31 | Male | 20.00 | 11.00 | No | 0.40 | 249.88 | 19.70 | 856.05 | 10.92 |
| 9 | 77 | Female | 73.00 | 4.00 | No | 4.30 | 379.18 | 19.18 | 755.24 | 12.74 |
| 10 | 48 | Female | 40.00 | 8.00 | Yes | 5.70 | 126.48 | 18.66 | 125.14 | 11.37 |
| 11 | 42 | Female | 37.00 | 5.00 | No | 3.20 | 191.55 | 17.63 | 386.53 | 13.21 |
| 12 | 55 | Female | 50.00 | 5.00 | No | 4.80 | 352.50 | 20.23 | 559.28 | 14.16 |
| 13 | 20 | Female | 10.00 | 10.00 | Yes | 2.90 | 432.55 | 19.70 | 491.45 | 15.62 |
| 14 | 34 | Male | 27.00 | 7.00 | No | 3.10 | 22.25 | 20.76 | 676.73 | 20.76 |
| 15 | 54 | Female | 34.00 | 20.00 | Yes | 3.70 | 205.32 | 20.76 | 93.29 | 16.62 |
| 16 | 25 | Male | 20.00 | 5.00 | No | 6.20 | 323.87 | 19.97 | 60.62 | 19.18 |
| 17 | 28 | Male | 12.00 | 16.00 | No | 0.30 | 627.01 | 20.50 | - | - |
| 18 | 45 | Male | 30.00 | 15.00 | No | 15.20 | 300.89 | 23.48 | - | - |
| 19 | 26 | Male | 24.00 | 2.00 | No | 4.20 | 226.11 | 21.30 | - | - |
| 20 | 66 | Male | 51.00 | 15.00 | Yes | 7.30 | 398.34 | 22.11 | - | - |
| 21 | 25 | Male | 24.00 | 1.00 | No | 2.80 | 339.77 | 21.30 | - | - |
| 22 | 45 | Male | 25.00 | 20.00 | Yes | 9.40 | 219.16 | 21.57 | - | - |
| 23 | 44 | Male | 22.00 | 22.00 | No | 2.40 | 522.10 | 21.03 | - | - |
| 24 | 46 | Male | 43.00 | 3.00 | No | 6.30 | 880.01 | 20.50 | - | - |
| 25 | 15 | Female | 13.00 | 2.00 | No | 4.10 | 109.15 | 24.60 | - | - |
| 26 | 26 | Male | 14.00 | 12.00 | Yes | 0.20 | 404.26 | 22.93 | - | - |
| 27 | 45 | Female | 41.00 | 4.00 | No | 0.20 | 791.86 | 23.48 | - | - |
| 28 | 31 | Male | 23.00 | 8.00 | No | 3.80 | 470.18 | 23.21 | - | - |
| 29 | 73 | Female | 69.00 | 4.00 | No | 1.10 | 464.13 | 22.93 | - | - |
| 30 | 37 | Male | 36.50 | 0.50 | No | 1.20 | 15.98 | 24.88 | - | - |
| 31 | 57 | Male | 30.00 | 27.00 | Yes | 2.20 | 32.38 | 20.76 | - | - |
| 32 | 27 | Male | 24.00 | 3.00 | No | 6.00 | 620.65 | 18.66 | - | - |
| 33 | 37 | Male | 36.50 | 0.50 | No | 0.30 | 631.78 | 20.23 | - | - |
| 34 | 17 | Male | 15.00 | 2.00 | No | 0.30 | 80.16 | 22.38 | - | - |
| 35 | 45 | Male | 35.00 | 20.00 | No | 0.50 | 50.29 | 22.93 | - | - |
| 36 | 13 | Male | 12.50 | 0.50 | No | 2.60 | 78.85 | 23.76 | - | - |
| 37 | 42 | Female | 27.00 | 15.00 | No | 3.10 | 370.38 | 23.76 | - | - |
| 38 | 24 | Male | 23.00 | 1.00 | No | 4.20 | 379.18 | 25.16 | - | - |
| 39 | 44 | Female | 43.00 | 1.00 | No | 2.80 | 88.02 | 19.18 | - | - |
| 40 | 18 | Female | 16.00 | 2.00 | No | 1.80 | 379.18 | 18.40 | - | - |
| 41 | 63 | Male | 55.00 | 8.00 | No | 4.00 | 485.36 | 20.50 | - | - |
| 42 | 38 | Male | 37.50 | 0.50 | No | 4.20 | 450.55 | 18.92 | - | - |
| 43 | 70 | Female | 68.00 | 2.00 | Yes | 1.20 | 503.68 | 19.44 | - | - |
