# Supplementary material for: Evaluation of Serum Proinflammatory Cytokine IL-17A and Tight Junction Protein Claudin-1 in Psoriasis
Source: Contrast Media Mol Imaging. 2022 Mar 7;2022:6092808. doi: 10.1155/2022/6092808 (PMC8920681; doi:10.1155/2022/6092808)
Supplement: Supplementary Materials — Supplementary Table 1: patients' demographic and clinical characteristics, as well as IL-17A and Claudin-1 serum levels. Supplementary Table 2: the values of serum assay IL-17A and Claudin-1 in psoriatic patients and controls. Supplementary Table 3: patients' demographic and clinical characteristics, as well as the serum levels of IL-17A and Claudin-1. Supplementary Table 4: correlation between serum IL-17A, Claudin-1, and other parameters in the psoriasis group and subgroups. Supplementary Table 5: diagnostic performances of serum IL-17A, Claudin-1, and IL-17A + Claudin-1 in psoriasis. Supplementary Material. [file 6092808.f1.zip › 6092808.f1/Supplementary Table 2 (1).doc]

| Supplementary Table 2.The values of serum assay IL-17A and claudin-1 in psoriatic patients and controls. | | | |
| --- | --- | --- | --- |
| Assay | Psoriatic patients (n=43) | Controls （n=16) | p values |
| Claudin-1(pg/mL) |  |  |  |
| Mean±SD | 327.00±33.00 | 538.82±85.12 |  |
| Median (min–max) | 339.78 (15.98-880.01) | 519.15 (60.62-1428.69) | **0.0063**^**^ |
| IL-17A (pg/ml) |  |  |  |
| Mean±SD | 20.88±0.31 | 13.12±0.88 |  |
| Median (min–max) | 20.50 (17.12-25.16) | 12.51 (7.52-20.76) | ＜0.0001^***^ |
| Mann-Whitney U test; **P < 0.001, ***P <0.0001. | | | |
