# Supplementary material for: Evaluation of Serum Proinflammatory Cytokine IL-17A and Tight Junction Protein Claudin-1 in Psoriasis
Source: Contrast Media Mol Imaging. 2022 Mar 7;2022:6092808. doi: 10.1155/2022/6092808 (PMC8920681; doi:10.1155/2022/6092808)
Supplement: Supplementary Materials — Supplementary Table 1: patients' demographic and clinical characteristics, as well as IL-17A and Claudin-1 serum levels. Supplementary Table 2: the values of serum assay IL-17A and Claudin-1 in psoriatic patients and controls. Supplementary Table 3: patients' demographic and clinical characteristics, as well as the serum levels of IL-17A and Claudin-1. Supplementary Table 4: correlation between serum IL-17A, Claudin-1, and other parameters in the psoriasis group and subgroups. Supplementary Table 5: diagnostic performances of serum IL-17A, Claudin-1, and IL-17A + Claudin-1 in psoriasis. Supplementary Material. [file 6092808.f1.zip › 6092808.f1/Supplementary Table 3 (1).doc]

| Supplementary Table 3.Patients’ demographic and clinical characteristics, as well as the serum levels of IL-17A and clauidn-1. | | | | | | | |
| --- | --- | --- | --- | --- | --- | --- | --- |
| Characteristics | Psoriasis | Early-onset psoriasis ((n=30) | Late-onset psoriasis (n=13) | *p* values | Mild psoriasis (n=17) | Moderate-severe psoriasis (n=26) | *p* values |
| Age (year) | 40.42±16.19 | 33.13±11.54 | 57.23±12.52 | **≤0.001***** | 36.59±16.53 | 42.92±15.78 | 0.214 |
| Gender (Female/Male) | 29/14 | 23/7 | 6/7 | 0.051 | 10/7 | 19/7 | 0.341 |
| Early/late onset (<40/>40) | 30/13 | 30 | 13 | - | 11/6 | 19/7 | 0.57 |
| Mild/moderate-severe psoriasis | 17/26 | 1.63±0.49 | 1.54±0.42 | 0.57 | 9/8 | 16/10 | - |
| Stages (Active/stable) | 25/18 | 16/14 | 9/4 | 0.344 | 17 | 26 | 0.587 |
| Disease duration (years) | 8.82±8.10 | 10.25±8.75 | 1.31±0.48 | 0.078 | 7.90±8.68 | 9.42±7.82 | 0.552 |
| Family history (Yes/No) | 9/34 | 5/25 | 4/9 | 0.308 | 9/43 | 34/43 | 0.742 |
| BSA (%) | 11.24±19.59 | 11.03±16.17 | 11.74±26.65 | 0.915 | 0.95±0.64 | 17.97±22.92 | **0.004**** |
| BMI ( kg/m^2^) | 24.25±3.32 | 23.63±3.43 | 25.68±2.66 | 0.062 | 23.40±2.90 | 24.80±3.52 | 0.18 |
| claudin-1 (pg/mL) | 327.04±216.42 | 270.73±175.54 | 456.98±251.49 | **0.008**** | 339.70±246.05 | 318.76±199.41 | 0.760 |
| IL-17A (pg/mL) | 20.88±2.04 | 21.06±2.23 | 20.45±1.49 | 0.37 | 20.66±1.83 | 21.02±2.19 | 0.576 |
| IL-2 (pg/mL) | 0.75±1.51 | 0.36±0.94 | 1.06±2.35 | 0.166 | 0.24±0.47 | 0.79±1.89 | 0.243 |
| IL-4 (pg/mL) | 0.15±0.24 | 0.11±0.75 | 0.22±0.43 | 0.182 | 0.10±0.00 | 0.18±0.31 | 0.315 |
| IL-6 (pg/mL) | 26.29±74.34 | 16.46±31.02 | 48.98±127.34 | 0.191 | 16.25±22.35 | 32.86±94.08 | 0.48 |
| IL-10 (pg/mL) | 0.85±2.87 | 0.45±0.93 | 1.77±5.04 | 0.171 | 1.51±4.51 | 4.82±0.56 | 0.227 |
| TNF-α (pg/mL) | 1.70±2.78 | 1.26±2.06 | 2.73±3.90 | 0.113 | 1.34±2.22 | 1.94±3.16 | 0.494 |
| IFN-γ (pg/mL) | 0.21±0.37 | 0.18±0.34 | 0.27±0.44 | 0.454 | 0.21±0.45 | 0.21±0.32 | 0.999 |
| CRP (pg/mL) | 4.20±7.18 | 4.85±8.39 | 2.70±2.57 | 0.371 | 1.93±2.31 | 5.69±8.79 | 0.093 |
| IgE (pg/mL) | 115.62±183.66 | 128.00±146.93 | 84.66±115.26 | 0.367 | 110.78±154.72 | 118.91±129.84 | 0.855 |
| Nail psoriasis (Yes/No) | 23/20 | 15/15 | 8/5 | 0.498 | 11/6 | 17/9 | 0.055 |
| Arthritis (Yes/No) | 8/35 | 5/25 | 3/10 | 0.63 | 14/3 | 4/22 | 0.514 |
| Comorbidity (Yes/No) | 32/11 | 10/20 | 12/1 | 0.08 | 15/2 | 17/9 | 0.097 |
| PASI (0–72) | 3.63±3.03 | 3.89±3.23 | 3.04±2.50 | 0.406 | 1.45±1.26 | 5.06±3.00 | **≤0.001***** |
| **P < 0.001, ***P <0.0001. | | | | | | | |
