# Supplementary material for: Evaluation of Serum Proinflammatory Cytokine IL-17A and Tight Junction Protein Claudin-1 in Psoriasis
Source: Contrast Media Mol Imaging. 2022 Mar 7;2022:6092808. doi: 10.1155/2022/6092808 (PMC8920681; doi:10.1155/2022/6092808)
Supplement: Supplementary Materials — Supplementary Table 1: patients' demographic and clinical characteristics, as well as IL-17A and Claudin-1 serum levels. Supplementary Table 2: the values of serum assay IL-17A and Claudin-1 in psoriatic patients and controls. Supplementary Table 3: patients' demographic and clinical characteristics, as well as the serum levels of IL-17A and Claudin-1. Supplementary Table 4: correlation between serum IL-17A, Claudin-1, and other parameters in the psoriasis group and subgroups. Supplementary Table 5: diagnostic performances of serum IL-17A, Claudin-1, and IL-17A + Claudin-1 in psoriasis. Supplementary Material. [file 6092808.f1.zip › 6092808.f1/Supplementary Table 4 (1).doc]

| Supplementary Table 4. Correlation between serum IL-17A, claudin-1 and other parameters in the psoriasis group and subgroups. | | | | | | | | | | |
| --- | --- | --- | --- | --- | --- | --- | --- | --- | --- | --- |
| Characteristics | Psoriasis (n=43) | | Early-onset psoriasis (n=30) | | Late-onset psoriasis (n=13) | | Mild psoriasis (n=17) | | Moderate-severe psoriasis(n=26) | |
|  | IL-17A | Claudin-1 | IL-17A | Claudin-1 | IL-17A | Claudin-1 | IL-17A | Claudin-1 | IL-17A | Claudin-1 |
|  | r(*p*) | r(*p*) | r(*p*) | r(*p*) | r(*p*) | r(*p*) | r(*p*) | r(*p*) | r(*p*) | r(*p*) |
| Age (year) | -0.131(0.401) | 0.178(0.254) | -0.118(0.536) | -0.166(0.382) | -0.081(0.802) | -0.126(0.697) | -0.290(0.259) | 0.231(0.373) | 0.193(0.345) | 0.246(0.226) |
| Gender (Female/Male) | -0.144(0.356) | -0.004(0.980) | -0.114(0.549) | -0.027(0.886) | -0.247(0.440) | -0.416(0.178) | -0.516(**0.034***) | 0.122(0.641) | -0.104(0.613) | -0.075(0.715) |
| Early/late onset (<40/>40) | -0.143(0.360) | 0.384(**0.011***) | - | - | - | - | -0.215(0.407) | 0.452(0.068) | 0.370(0.063) | 0.410(**0.037***) |
| Mild/moderate-severe | 0.090(0.565) | -0.031(0.845) | 0.084(0.659) | 0.016(0.933) | 0.097(0.764) | -0.145(0.653) | - | - | - | - |
| Stages (Active/stable) | -0.049(0.753) | -0.034(0.828) | -0.189(0.316) | 0.054(0.777) | 0.258(0.418) | -0.051(0.874) | 0.291(0.257) | -0.048(0.855) | -0.063(0.759) | 0.242(0.233) |
| Disease duration (years) | -0.027(0.865) | -0.16(0.306) | -0.175(0.355) | -0.010(0.960) | 0.048(0.882) | -0.448(0.144) | 0.082(0.753) | -0.269(0.296) | -0.080(0.696) | -0.078(0.707) |
| Family history (Yes/No) | -0.030(0.849) | -0.069(0.660) | 0.047(0.807) | -0.005(0.978) | 0.130(0.687) | 0.028(0.931) | -0.128(0.624) | 0.170(0.515) | -0.293(0.147) | 0.072(0.728) |
| BSA (%) | -0.004(0.979) | 0.073(0.644) | -0.065(0.732) | 0.160(0.398) | -0.505(0.094) | -0.362(0.247) | 0.387(0.125) | 0.136(0.603) | 0.148(0.470) | 0.049(0.811) |
| BMI ( kg/m^2^) | 0.011(0.946) | 0.173(0.267) | 0.024(0.899) | 0.126(0.506) | 0.123(0.703) | -0.056(0.863) | 0.331(0.195) | -0.034(0.896) | 0.087(0.671) | 0.021(0.921) |
| Claudin-1 (pg/mL) | -0.081(0.607) | 1.000(≤0.001) | -0.196(0.300) | 1.000(≤0.001) | 0.630(**0.028***) | 1.000(≤0.001) | -0.120(0.647) | 1.000(≤0.001) | -0.041(0.844) | 1.000(≤0.001) |
| IL-17A (pg/mL) | 1.000(≤0.001) | -0.081(0.607) | 1.000(≤0.001) | -0.196(0.300) | 1.000(≤0.001) | 0.630(**0.028***) | 1.000(≤0.001) | -0.120(0.647) | 1.000(≤0.001) | -0.041(0.844) |
| IL-2 (pg/mL) | -0.036(0.819) | 0.177(0.256) | 0.104(0.586) | 0.278(0.137) | -0.098(0.762) | 0.047(0.885) | 0.148(0.572) | 0.162(0.535) | -0.041(0.844) | -0.106(0.607) |
| IL-4 (pg/mL) | -0.178(0.252) | -0.009(0.957) | -0.161(0.395) | 0.118(0.535) | -0.609(0.036*) | -0.371(0.235) | NS | NS | 0.267(0.187) | 0.062(0.762) |
| IL-6 (pg/mL) | -0.397(**0.008****) | 0.024(0.881) | -0.324(0.080) | 0.034(0.859) | -0.674(0.016*) | -0.113(0.727) | -0.513(**0.035***) | 0.267(0.301) | -0.002(0.993) | 0.178(0.384) |
| IL-10 (pg/mL) | -0.015(0.925) | -0.032(0.841) | 0.177(0.349) | -0.128(0.499) | -0.376(0.228) | 0.036(0.911) | 0.055(0.834) | -0.075(0.776) | -0.094(0.649) | 0.018(0.929) |
| TNF-α (pg/mL) | -0.425(**0.005****) | 0.204(0.189) | -0.364(**0.048***) | 0.206(0.275) | -0.342(0.276) | 0.299(0.346) | -0.383(0.129) | 0.306(0.232) | 0.009(0.965) | -0.041(0.841) |
| IFN-γ (pg/mL) | 0.129(0.410) | -0.128(0.412) | 0.183(0.333) | -0.201(0.287) | -0.352(0.262) | -0.131(0.685) | 0.026(0.922) | -0.255(0.323) | 0.128(0.533) | 0.289(0.153) |
| CRP (pg/mL) | -0.063(0.686) | 0.056(0.720) | -0.081(0.671) | 0.149(0.432) | 0.007(0.983) | -0.392(0.208) | -0.141(0.588) | 0.097(0.711) | -0.014(0.946) | 0.045(0.826) |
| IgE (pg/mL) | 0.149(0.340) | 0.089(0.571) | 0.148(0.435) | 0.153(0.419) | 0.039(0.905) | 0.252(0.430) | -0.065(0.803) | -0.047(0.859) | 0.014(0.944) | 0.003(0.988) |
| Nail psoriasis (Yes/No) | -0.246(0.111) | 0.068(0.666) | -0.185(0.327) | 0.154(0.416) | -0.493(0.103) | -0.318(0.313) | -0.367(0.147) | 0.025(0.924) | 0.178(0.385) | 0.210(0.303) |
| Arthritis (Yes/No) | 0.034(0.830) | 0.12(0.442) | 0.191(0.311) | 0.036(0.849) | -0.421(0.173) | 0.195(0.543) | -0.200(0.442) | 0.396(0.115) | -0.178(0.385) | -0.441(**0.024***) |
| Comorbidity (Yes/No) | -0.144(0.357) | -0.026(0.870) | -0.147(0.437) | -0.127(0.505) | -0.264(0.407) | -0.480(0.114) | -0.225(0.385) | 0.112(0.669) | -0.065(0.754) | 0.275(0.174) |
| PASI (0–72) | -0.199(0.202) | -0.026(0.867) | -0.181(0.339) | 0.141(0.451) | -0.355(0.257) | -0.204(0.526) | -0.464(0.060) | -0.350(0.169) | -0.322(0.109) | 0.174(0.395) |
| r, Spearman’s correlation coefficient; *p*, significance; *, p<0.05; **, p<0.01; NS, not available. | | | | | | | | | | |
