# Supplementary material for: Evaluation of Serum Proinflammatory Cytokine IL-17A and Tight Junction Protein Claudin-1 in Psoriasis
Source: Contrast Media Mol Imaging. 2022 Mar 7;2022:6092808. doi: 10.1155/2022/6092808 (PMC8920681; doi:10.1155/2022/6092808)
Supplement: Supplementary Materials — Supplementary Table 1: patients' demographic and clinical characteristics, as well as IL-17A and Claudin-1 serum levels. Supplementary Table 2: the values of serum assay IL-17A and Claudin-1 in psoriatic patients and controls. Supplementary Table 3: patients' demographic and clinical characteristics, as well as the serum levels of IL-17A and Claudin-1. Supplementary Table 4: correlation between serum IL-17A, Claudin-1, and other parameters in the psoriasis group and subgroups. Supplementary Table 5: diagnostic performances of serum IL-17A, Claudin-1, and IL-17A + Claudin-1 in psoriasis. Supplementary Material. [file 6092808.f1.zip › 6092808.f1/Supplementary Table 5 (1).doc]

| Supplementary Table 5.Diagnostic performances of serum IL-17A, claudin-1 and IL-17A+ claudin-1 in psoriasis. | | | | | | | | |
| --- | --- | --- | --- | --- | --- | --- | --- | --- |
| Group | Method | Cut-off | Sensitivity(95%CI) | Specificity(95%CI) | LR+ | LR- | AUC(95%CI) | P value |
| Psoriasis/controls | IL-17A | 16.620 | 100.00%(91.8%-100.0%) | 87.50%(61.7%-98.4%) | 8.00 | 0.00 | 0.951(0.861-0.990) | **<0.0001***** |
|  | Claudin-1 | 379.178 | 65.12%(49.1%-79.0%) | 75.00%(47.6%-92.7%) | 2.60 | 0.47 | 0.709(0.577-0.820) | **0.0119*** |
|  | Combination | 0.498 | 100.00%(91.8%-100.0%) | 87.50%(61.7%-98.4%) | 8.00 | 0.00 | 0.949(0.858-0.989) | **<0.0001***** |
| Early/late-onset psoriasis | IL-17A | 20.496 | 76.92%(46.2%-95.0%) | 56.67%(37.4%-74.5%) | 1.78 | 0.41 | 0.590(0.429±0.737) | 0.3126 |
|  | Claudin-1 | 450.553 | 53.85%(25.1%-80.8%) | 86.67%(69.3%-96.2%) | 4.04 | 0.53 | 0.741(0.585±0.862) | **0.0067**** |
|  | Combination | 0.263 | 76.92%(46.2%-95.0%) | 66.67%(47.2%-82.7%) | 2.31 | 0.35 | 0.741(0.585±0.862 | **0.0045**** |
| Mild/moderate-severe psoriasis | IL-17A | 20.230 | 61.54%(40.6%-79.8%) | 58.82%(32.9%-81.6%) | 1.49 | 0.65 | 0.553(0.394-0.705) | 0.5596 |
|  | Claudin-1 | 620.645 | 96.15%(80.4%-99.9%) | 23.53%(6.8%-49.9%) | 1.26 | 0.16 | 0.518(0.361-0.673) | 0.8539 |
|  | Combination | 0.615 | 46.15%(26.6%-66.6%) | 70.59%(44.0%-89.7%) | 1.57 | 0.76 | 0.559(0.399-0.710) | 0.5225 |
| *, p<0.05;**, p<0.01; ***<0.001 | | | | | | | | |
